# Utility of diffusion weighted Magnetic Resonance Imagining to detect non-palpable undescended testis

**DOI:** 10.12669/pjms.40.9.8905

**Published:** 2024-10

**Authors:** Nasreen Naz, Ayesha Walid, Anila Rahim, Komal Lajpat

**Affiliations:** 1 Nasreen Naz, FCPS, Department of Radiology, DUHS, Ojha Campus, Karachi, Pakistan; 2 Ayesha Walid, FCPS, Department of Radiology, DUHS, Ojha Campus, Karachi, Pakistan; 3 Anila Rahim, MD, Radiology, Department of Radiology, DUHS, Ojha Campus, Karachi, Pakistan; 4 Komal Lajpat, MBBS, FCPS. Civil Hospital Karachi (CHK), Dow University of Health Sciences (DUHS) Karachi, Pakistan

**Keywords:** Non-palpable Undescended Testis (NPUT), Undescended Testis, Diffusion-Weighted Magnetic Resonance Imaging (DW-MRI)

## Abstract

**Objective:**

To assess the effectiveness of diffusion-weighted magnetic resonance imaging (DW-MRI) in the detection of non-palpable undescended testis (NPUT) and to compare DW-MRI results with surgical findings.

**Methods:**

This descriptive, cross-sectional study was carried out at Dow Institute of Radiology, Dow University of Health Sciences. Karachi on a cohort of patients who underwent DW-MRI for suspected NPUT between 15^th^ September, 2022 to 16^th^ March, 2023. The study included 175 boys below the age of 16 years with history of clinically non-palpable testes. MRI scans were acquired using additional DWI sequence. Two radiology faculty of more than five years of experience independently evaluated the DW-MRI images for the presence or absence of testes in its normal anatomical position, abnormal location, side and size of testes.

**Results:**

Out of the 175 patients, DW-MRI successfully detected 128 (73.1%) non-palpable undescended testes. Statistical analysis in clinically detected cases of showed sensitivity of NPTU 0.8%, specificity of 92.5%, diagnostic accuracy 94.29%, positive predictive value 97.7%, and negative predictive value 84.1% for localizing undescended testes with Diffusion Weighted DW-MRI taking surgical findings as gold standard.

**Conclusion:**

DWI sequences complement the conventional MRI, increasing its sensitivity and diagnostic accuracy. By facilitating early and accurate diagnosis, DW-MRI has the potential to streamline patient management, reduce unnecessary surgical exploration, and ultimately improve the long-term reproductive health and quality of life for individuals with undescended testes.

## INTRODUCTION

The failure of one or both testicles to descend into the scrotum from the abdomen, where they develop before birth, is known as cryptorchidism or undescended testis.^1^ These are usually spontaneously corrected by the age,^1^ with the exception of 0.8% of boys.^2^ Undescended testicles are found in 21% in term boys and 1.8-4.0% in preterm neonates.^3^ Physical examination reveals that 80% of the undescended testes are clinically appreciable.^2^ Non-palpable testicles might be missing (45%), atrophic (45%), or situated in the belly (50%) or inguinal canal (5%).^4^,^5^ Cryptorchidism has been associated with infertility, inguinal hernias, and an increased risk of testicular cancer.^6^ Preoperative imaging examination for accurate diagnosis of non-palpable viable testis in determining the best surgical approach is critical. Surgical exploration is not required if the testes are missing or atrophic on MRI.^7^

Various imaging modalities have been utilized for the identification of non-palpable testis, each with their own set of limitations. Ultrasound is the most easily available, least expensive modality, that can help locate the testes within the inguinal canal however, missing out intra-abdominal testes. Furthermore, ultrasound accuracy is contingent on the operator’s expertise especially in obese and uncooperative children.^8^ According to literature reports, ultrasound imaging has low sensitivity as compared to MRI.^9^ Using a CT scan, the non-palpable testis can be differentiated from the cord’s contents and swollen lymph nodes.^10^ The main disadvantage of CT, on the other hand, is the associated radiation danger to the testes. CT has never been shown to be able to identify raised abdominal testis.^5^ The most precise diagnostic technique to identify non-palpable testes has been is laparoscopy which is, however, intrusive.^5^,^11^

MRI allows tissue characterization and multi-planar imaging while being noninvasive and radiation-free.^5^,^7^,^12^,^13^ Standard MRI has demonstrated low specificity and intermediate sensitivity for the evaluation of non-palpable testes, which makes it less effective for making a decision for surgical laparoscopy.^7^,^13^ Diffusion-weighted imaging and fat-suppressed T2 weighted imaging are great sequences for detecting cryptorchidism. Lymph nodes and testes can be distinguished using fat-suppressed T2WI, which is incredibly sensitive to inflammation and water content.^7^ The testicular tissues’ functional and structural information is provided by the DWI sequence, which enables tissue characterization.^4^-^7^

International literature has proven the role of DWI as adjunct to conventional MRI to increase its diagnostic precision. In developing countries generally surgical intervention is undertaken without pre-operative MRI due to limited resource available and cost affordability This study will assess the utility of DWI sequence in MRI for diagnosis of undescended testis and will co relate the results with surgical findings with a view to minimize the number of unnecessary surgical interventions and compare the results with surgical findings.

## METHODS

This descriptive, cross-sectional study was carried out at Dow Institute of Radiology DUHS, Karachi from 15^th^ September, 2022 to 16^th^ March, 2023.Non-probability-consecutive sampling technique was used. This study included boys under the age of 16 with non-palpable undescended testis (NPUT) referred for MRI by the surgeons and clinicians. Those having pre-term birth weight, ambiguous genitalia, prior lower abdominal, scrotal, or anorectal surgery or with renal malformations, were excluded from the study. Non-consensual and lost data were also excluded from the study. Demographic information was noted after receiving written informed consent from the parents and guardian. Data anonymity and confidentiality maintained by generating specific password.

### Ethical Approval:

It was obtained from the IRB. (Ref. No.: IRB 2621/DUHS/Approval/2022/1032).

All the MRI were procedures were conducted on a 1.5-T closed configuration system (GE Medical System). Chloral hydrate at a dose of 1 ml/kg body weight was used to sedate the boys under the age of five. The MRI examination was conducted with a body coil in supine position and their head oriented in the direction of the magnet. The abdomen was covered, below from the scrotal region and above to the renal region. Straps held the body coil in place to prevent respiratory artifacts. Different MRI sequences used that included free breathing axial and coronal spin-echo T1-weighted sequence, axial T2- weighted sequence, axial and coronal fat suppressed spin-echo T2-weighted sequence and axial DWI. Diffusion Weighted Images were performed with three sets of p value of 50, 400, and 800 s/mm 2, each with acquisition time of three minutes.

**Fig.1:**
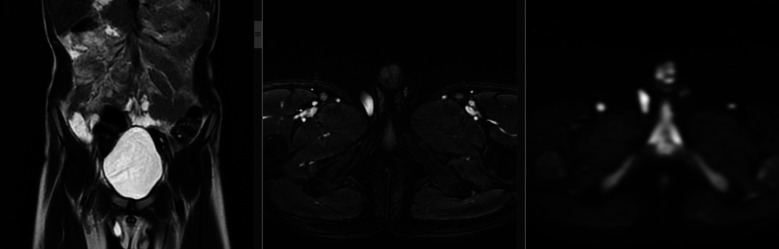
7-year-old boy presenting with right inguinal testis on MRI, appears of high signal intensity. On (a) coronal T2W, (b) axial T2W fat - sat, and (c) axial DWI.

**Fig.2:**
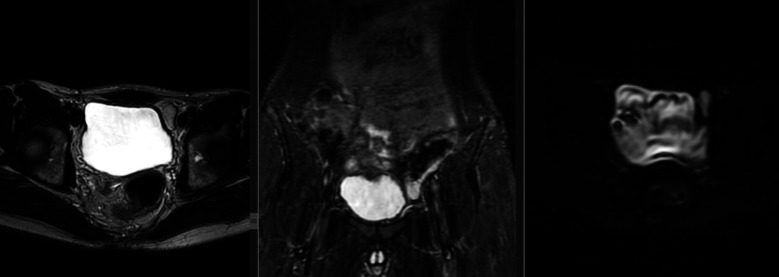
MRI localized testis in left low intra - abdominal location, as shown on (a) axial T2W, (b) coronal T2W fat - sat, and (c) axial DWI.

All the MRI images were assessed and reported by radiology faculty with at least five years of expertise in reporting abdominal MRI. Radiologist recorded the presence or absence of testis and its location. On MR images, testes appear as elliptic areas of low signal intensity on T1WI and hyper-intense on T2WI. These also appear hyper-intense on DWI due to their high cellular content. Therefore, testes were identified as localized ovoid areas of hyper-intensity on DWI that did not correspond to T2 shine-through from fluid-containing structures. Their anatomic location was classified as: intra canalicular (Testis close to and below the internal inguinal ring) (Fig.1), low intra-abdominal (Testis above the internal ring, seen around the iliac vessels) (Fig.2), and high intra-abdominal (Testis located more than 3cm above the internal ring).

All participants had laparoscopic exploration within two weeks of MRI. MRI findings were compared to laparoscopic outcome, which was taken as a gold standard. All laparoscopic examinations were performed by the same surgeon to eliminate bias. The results of MRI were considered positive when testes identified undescended both on MRI and on laparoscopic findings. NPV…PPV.

### Statistical Analysis:

Data was analyzed using SPSS version 23.0. Mean and standard deviation was calculated for quantitative data as age and side affected. Qualitative variables from MRI findings as testis localized / not localized and the position of undescended testis were expressed as frequency and percentage. The sensitivity, specificity, PPV, NPV, and diagnostic accuracy of DW-MRI imaging in the identification of undescended testis were calculated using a 2x2 contingency table.

## RESULTS

The 175 boys with clinically undescended testes ranged from six months to 16 years (Mean = 6.8 ± 2.8 years). All boys with normal range birth weight were taken as study population with mean birth weight of 3.4 ± 1.6 kg.

Distribution of undescended testis showed right side for 132 (75.4%) while left side for 43 (24.6%) patients. Total 131 testes (74.8%) were detected on DW-MRI while surgery localized 135 (77.1%) testes (Table-I). DW-MRI diagnosis of undescended testes was confirmed in 128 (73.1%) patients on laparoscopy (true positive). Three testes localized by DWI=MRI were not confirmed with laparoscopy (false positive). Out of 175 boys, Diffusion Weighted MR images failed to detect seven testes, which were localized by laparoscopy giving false negative results. Four of these were atrophic and localized in high intra- abdominal position. Thirty seven testes could not be identified by both examinations, i.e., DWI-MRI and laparoscopy; likely absent, these were considered true negative. (Table-II).

**Table-I:** Location of undescended testis detected on DW-MRI and surgery.

DW-MRI N = 131		Surgery N = 135		

Location	No.	%	No.	%
Intra canalicular	73	55.7	73	54
Low intra-abdominal	35	26.7	35	25.9
High intra-abdominal	23	17.7	27	20

**Table-II:** Comparison of DW-MRI and laparoscopic findings.

MRI	Surgery		Total

	Positive	Negative	
Positive	128 (True positive)	3 (False positive)	131
Negative	7 (False negative)	37 (True negative)	44

Total	135	40	175

Statistical analysis in clinically detected cases of NPUT showed sensitivity of 94.8%, specificity of 92.5%, diagnostic accuracy 94.29%, positive predictive value 97.7%, and negative predictive value 84.1% for localizing undescended testes.

## DISCUSSION

Laparoscopy is the most efficient and accurate method for identifying non-palpable testes; however, the main drawback of this method is that it is intrusive.^14^,^15^ While MRI does an adequate task of recognizing inguinoscrotal areas, it is less accurate at locating most atrophied testicles, making it a less reliable approach for identifying which children require surgery and which ones do not. Traditional MRI had a reported sensitivity of 55% for finding intra-abdominal testicles and 86% for inguino-scrotal testicles.^16^ In our study additional sequences as T2-weighted fat-suppressed and diffusion weighted MRI were added to conventional sequencing, that showed an increased sensitivity of 94.8 % with improved diagnostic accuracy.

The T2W Fat-suppression sequencing is more responsive to inflammation and water content, which allows for a clear distinction between the testes and the surrounding tissues.^13^,^17^ This sequence is very effective in differentiating lymph nodes from testicles. Diffusion-weighted in conjunction with traditional MRI sequences, enables a better characterization of the tissue. Furthermore, in cases of absent/vanishing testis, it prevents the need of unnecessary surgical exploration. We acquired diffusion-weighted images for our study at different ranges of b values 50,400,800 s/mm 2. At low b value fluid have high signal intensity in images. That’s why fluid containing structures, such as the colon, urine bladder, and gall bladder, have high signal intensity in images. To suppress the bowel high b values were selected. Testes are simple to localize in images with strong signal intensity at a b value of 800 s/mm2. Hence, similar to Mohammed et al.^13^, we also recommend the use of DWI at high b value for accurate testicular localization.

In this study, the mean age was 6.8 ± 2.8 years, which is quite comparable to studies of Ali et al. and Mansour et al.^18^ who reported mean age 6.4 years. Another study by Naseem et al. 19, reports mean age for diagnosis of cryptorchidism as 10.37 +/- 5.66 years. Recent study by K. Fazal et al.^14^, reports mean age at 17.08 ± 7.99 years, because they included patients with much larger age range. Age at time of diagnosis is an important prognostic factor, as risk of testicular malignancy and infertility increases with age.

Our study cryptorchidism showed right sided predominance (75.4%) while 24.6 % had left undescended testes. Comparable result of right (76.5%) and left (23.5%) distribution of undescended testes has been reported by Tseng et al in Taiwan^20^ while Sheikh et al also reported similar frequency of right (78.9%) and left (21.1%) undescended testes in a study at Lahore.^21^

In our study we report sensitivity of 94.8%, specificity of 92.5% and diagnostic accuracy of 94.29% for DW-MRI in localizing undescended testis. Emad-Eldin et al.^22^ who reported that the sensitivity and diagnostic accuracy of conventional MRI increases from 90 to 93 % and 91 to 95.7 % respectively, when DWI sequences are included, have published similar results. Additionally, a study based on the Pakistani population stated a combined DWI and MRI sensitivity of 92.86%, specificity of 100%, NPV of 75% and PPV of 100% with accuracy of 94.12%.^23^ Recent study by Mohammed et al.^13^ also reports that high b value DWI increases the diagnostic accuracy of conventional MRI from 88.3% to 94.1%.

Naseem et al.^19^ investigated the role of DWI-MRI in children with undescended testis in Pakistan, and has published similar results, reporting sensitivity, specificity, positive predictive value, negative predictive value and diagnostic accuracy as 82 %, 86 %, 93 %, 89 %, and 78 % respectively. Recently published study by K. Fazal et al.^14^ however reports relatively lower sensitivity and accuracy of DWI-MRI as 65.1%, and 81.3% respectively.

Increasing availability of DW-MRI along with non invasive and radiation free nature favors its preferred use in future practice. Further directions for research include larger prospective studies to validate the findings and investigate long-term outcomes following DW-MRI-guided management of NPUT

### Limitations:

It includes single Centre study and small sample size. Other limitation of the study did not correlate the testicular viability on DWI with surgical findings, which warrants further investigation.

## CONCLUSION

DWI sequences complement the conventional MRI, increasing its sensitivity and diagnostic accuracy. By facilitating early and accurate diagnosis, DW-MRI has the potential to streamline patient management, reduce unnecessary surgical exploration, and ultimately improve the long-term reproductive health and quality of life for individuals with undescended testes.

### Recommendations:

Multi Centre study with a larger cohort of the patient and also correlate the testicular viability on DW-MRI with surgical findings that will helpful for further decision of surgical procedures findings to develop generalizability. It should therefore be integrated into the diagnostic algorithm for non-palpable undescended testis, particularly in cases where clinical examination is inconclusive or traditional imaging modalities yield equivocal results.

### Authors Contribution:

**NN:** Conceived, designed and ensure integrity of the research.

**AW:** manuscript writing. Data interpretation, Review

**AR:** Data collection, Review, involved in manuscript writing.

**KL:** statistical analysis. Review, data analysis

All authors are responsible and accountable for the accuracy and integrity of the work and gave final approval of manuscript.
